# THZ1 targeting CDK7 suppresses STAT transcriptional activity and sensitizes T-cell lymphomas to BCL2 inhibitors

**DOI:** 10.1038/ncomms14290

**Published:** 2017-01-30

**Authors:** Florencia Cayrol, Pannee Praditsuktavorn, Tharu M. Fernando, Nicholas Kwiatkowski, Rosella Marullo, M. Nieves Calvo-Vidal, Jude Phillip, Benet Pera, Shao Ning Yang, Kaipol Takpradit, Lidia Roman, Marcello Gaudiano, Ramona Crescenzo, Jia Ruan, Giorgio Inghirami, Tinghu Zhang, Graciela Cremaschi, Nathanael S. Gray, Leandro Cerchietti

**Affiliations:** 1 Department of Medicine, Hematology and Oncology Division, Weill Cornell Medicine, New York, New York 10065, USA; 2 Neuroimmunomodulation and Molecular Oncology Department, Institute for Biomedical Research (BIOMED), National Research Council of Argentina (CONICET), Catholic University of Argentina (UCA), C1107AFB Ciudad Autonoma de Buenos Aires, Argentina; 3 Department of Cancer Biology, Dana-Farber Cancer Institute, Department of Biological Chemistry and Molecular Pharmacology, Harvard Medical School, Boston, Massachusetts 02115, USA; 4 Department of Pathology and Laboratory Medicine, Weill Cornell Medicine, New York, New York 10065, USA

## Abstract

Peripheral T-cell lymphomas (PTCL) are aggressive diseases with poor response to chemotherapy and dismal survival. Identification of effective strategies to target PTCL biology represents an urgent need. Here we report that PTCL are sensitive to transcription-targeting drugs, and, in particular, to THZ1, a covalent inhibitor of cyclin-dependent kinase 7 (CDK7). The STAT-signalling pathway is highly vulnerable to THZ1 even in PTCL cells that carry the activating *STAT3* mutation Y640F. In mutant cells, CDK7 inhibition decreases STAT3 chromatin binding and expression of highly transcribed target genes like *MYC, PIM1, MCL1, CD30, IL2RA, CDC25A* and *IL4R*. In surviving cells, THZ1 decreases the expression of STAT-regulated anti-apoptotic BH3 family members MCL1 and BCL-XL sensitizing PTCL cells to BH3 mimetic drugs. Accordingly, the combination of THZ1 and the BH3 mimetic obatoclax improves lymphoma growth control in a primary PTCL *ex vivo* culture and in two STAT3-mutant PTCL xenografts, delineating a potential targeted agent-based therapeutic option for these patients.

## Abstract (web summary)

T-cell lymphomas are aggressive diseases associated with poor outcome. Here, the authors show that the THZ1, a CDK7 inhibitor, suppresses STAT transcriptional activity leading to apoptosis and sensitization to BCL2 inhibitors in T-cell lymphomas.

In contrast to anaplastic large cell lymphomas (ALCL) with characteristic anaplastic lymphoma kinase (ALK) translocation (ALCL-ALK^pos^), for which kinase inhibitors designed to target ALK have been developed^1^, most peripheral T-cell lymphomas (PTCL) are usually treated similarly with a combination of chemotherapy agents, commonly cyclophosphamide, doxorubicin, vincristine and prednisone (CHOP)^2^. According to the international T-cell lymphoma project analysis, the 5-year failure-free survival rates for PTLC-not otherwise specified (NOS), angioimmunoblastic T-cell lymphoma and ALCL-ALK^neg^ patients were only 20, 18 and 36%, respectively, following CHOP-based therapy^3^. These data clearly indicate that new agents are urgently needed to improve disease management and patient survival.

It is now largely accepted that PTCL represents a phenotypically heterogeneous group of malignancies that harbour a diverse array of molecular abnormalities many of unknown functional relevance^4^. This molecular and phenotypic heterogeneity increases as PTCLs evolve into an even more complex disease under the pressure of external factors from the microenvironment^5^,^6^ and as consequence of their intrinsic instability. In this scenario, we have speculated that a therapeutic approach that targets a common feature preferentially used by several oncogenic drivers could cripple tumours and help lead to their ultimate eradication. To identify potential broad therapeutic pathways and antineoplastic agents that can be translated to the clinic, we conducted an unbiased cell-based screening of FDA-approved drugs in PTCL-NOS, ALCL-ALK^neg^, ALCL-ALK^pos^ and Sezary Syndrome cell lines.

Here we report that inhibitors of the proteasome, RNA polymerase II (RNA POL2)-mediated transcription and lysine deacetylases (KDAC) exhibit broad antiproliferative potency across aggressive PTCL subtypes. Among the compounds affecting the activity of RNA POL2, the covalent inhibitor of cyclin-dependent kinase 7 (CDK7) THZ1, reduces RNA POL2-mediated gene transcription showing activity across representative PTCL cell lines. We demonstrate that CDK7 activity is necessary to maintain the transcriptional program induced by signal transducer and activator of transcription (STAT) proteins that are activated both aberrantly by mutation and by extracellular cues. *MYC, MCL1* and *BCL2L1* (BCL-XL) are among the downstream genes transcriptionally regulated by the CDK7-STAT axis. This mechanism is rationalized to develop a novel therapeutic combination of THZ1 with BH3 mimetic compounds in PTCL pre-clinical models.

## Results

### Cell-based screening assay identifies active compounds in PTCL

**Figure 1:**
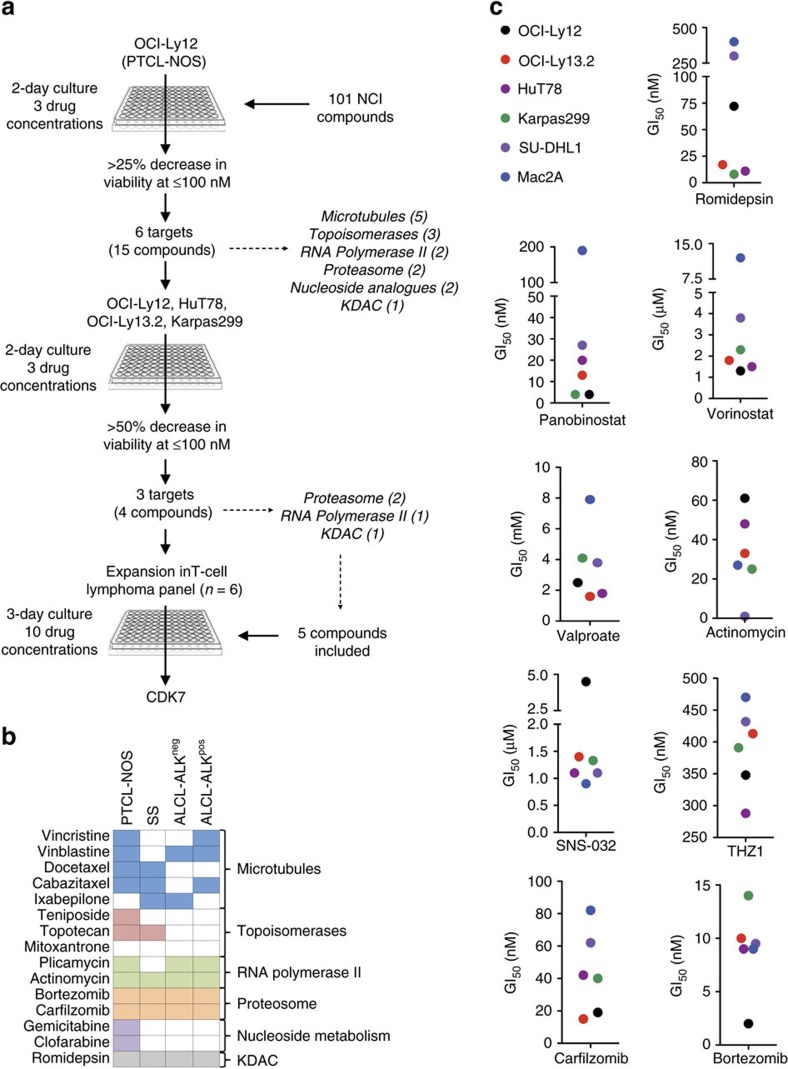
Screen for antineoplastic drugs and therapeutic targets in PTCL cell lines. (**a**) Screen setup and results. (**b**) Extended target validation in four PTCL cell lines (top). Drugs that showed >50% decrease in viability at <100 nM are shown as filled squares. (**c**) Growth inhibitory concentration 50% (GI_50_, *Y* axis) for nine compounds (shown on bottom) in an extended panel of six PTCL cell lines.

To identify therapeutic targets and potential drugs to translate to PTCL patients, we first screened a library of 101 known anti-neoplastic pharmacological agents (Supplementary Table 1) using the prototypical PTCL-NOS cell line OCI-Ly12 (Fig. 1a). Cells were exposed to drugs at three concentrations (10 and 1 μM, and 100 nM) for 48 h and analysed for inhibition of proliferation using a metabolic-based assay. A proliferation inhibition index was determined for each drug by comparing the mean effect in triplicates versus vehicle (DMSO or phosphate-buffered saline). We identified fifteen agents belonging to six targets that decrease OCI-Ly12 cell proliferation by 25% or more at 100 nM (Fig. 1a; Supplementary Table 1). To determine whether these pathways constitute therapeutic targets across the spectrum of PTCLs, we then tested these compounds in additional PTCL cell lines including HuT78 (Sezary Syndrome), OCI-Ly13.2 (ALK^neg^ ALCL) and Karpas299 (ALK^pos^ ALCL) using the same conditions as before. We identified four agents, bortezomib, carfilzomib, actinomycin and romidepsin, which decreased the proliferation of all cell lines tested by 50% or more at 100 nM (Fig. 1a,b). These four drugs inhibit three main pathways: romidepsin is a class I KDAC inhibitor, bortezomib and carfilzomib are proteasome inhibitors and actinomycin binds to the premelted DNA conformation at the transcription initiation complex and prevents the elongation of RNA chain by the RNA polymerase (RNA POL)^7^ (Fig. 1a,b).

We then expanded the screening to include additional compounds targeting the KDAC (panobinostat, vorinostat and valproate) and transcription/RNA POL-2, (SNS-032 (ref. 8) and THZ1 (ref. 9)) pathways as well as incorporating additional PTCL cell lines Mac2A (ALCL-ALK^neg^) and SU-DHL1 (ALCL-ALK^pos^). Cells were treated in dose–response for 3 days and growth inhibitory concentrations (GI_50_) were calculated. Most of the targets were validated in the complete panel of six PTCL cell lines, with actinomycin and THZ1 for transcription/RNA POL-2 and carfilzomib and bortezomib for proteasome showing activity in the nanomolar range across the spectrum of the disease (Fig. 1c). Romidepsin was the most potent KDAC inhibitor (GI_50_<75 nM), with two ALCL cell lines, Mac2A and SU-DHL1, showing relative resistance to the drug (GI_50_>250 nM, Fig. 1c). For drugs with available human or mouse pharmacokinetic data, we compared their GI_50_ *in vitro* with their *C*_max_ (maximum concentration) in blood to determine the range of potential clinical activity. All the drugs tested showed activity within the clinical-range limit except valproate and SNS-032 for all cell lines and panobinostat and vorinostat for Mac2A.

These results are in agreement with recent clinical data showing that proteasome inhibitors (that is, bortezomib^10^) and KDAC inhibitors (that is, romidepsin^11^, belinostat^12^) are compounds with activity in PTCL yielding about 25% of overall responses^13^. To explore new therapeutic pathways, we focused in transcriptional inhibitors for further development. Among other activities, actinomycin inhibits the transcription initiation complex and prevents RNA elongation by RNA POL2; given this broad mechanism of action, it is associated with serious side effects that limit its clinical use either alone or in combination. THZ1, that inhibits CDK7 at lower concentrations and CDK12 at higher concentrations, possesses a favourable pre-clinical toxicity profile and exhibits efficacy in several mouse xenograft models^9^,^14^,^15^. The phosphorylation of the C-terminal domain of RNA POL2 at the Ser5 by the CDK7 kinase subunit of transcription factor II human (TFIIH) accompanies the transition from transcription initiation to early elongation^16^,^17^. THZ1 covalently binds to the Cys312 residue of CDK7 decreasing Ser5-RNA POL2 (ref. 9). To determine the specificity of THZ1 towards CDK7, we expressed CDK7^WT^ and the CDK7^C312S^ mutant in 293T cells and exposed them to several concentrations of THZ1 for 48 h. This mutation prevented THZ1 from inhibiting CDK7 (Supplementary Fig. 1a) therefore causing an increase of about 6 times in its growth inhibitory concentration 50% (GI_50_, to 1,250 nM in CDK7^C312S^ from 210 nM in CDK7^WT^, Supplementary Fig. 1b).

To validate the inhibition of CDK7 as therapeutic target in PTCL cell lines, we used THZ1-R, a THZ1 analog with no significant inhibitory activity of CDK7 in cells^9^. In triplicate experiments, the mean GI_50_ (±95% CI) for THZ1 and THZ1-R in our panel of PTCL cell lines were 390±26 nM and 5365±360 nM, respectively (*P*<0.0001, *T*-test, Supplementary Fig. 1c), suggesting that CDK7 could be a relevant target in PTCLs.

### The covalent CDK7 inhibitor THZ1 induces apoptosis in PTCL

**Figure 2:**
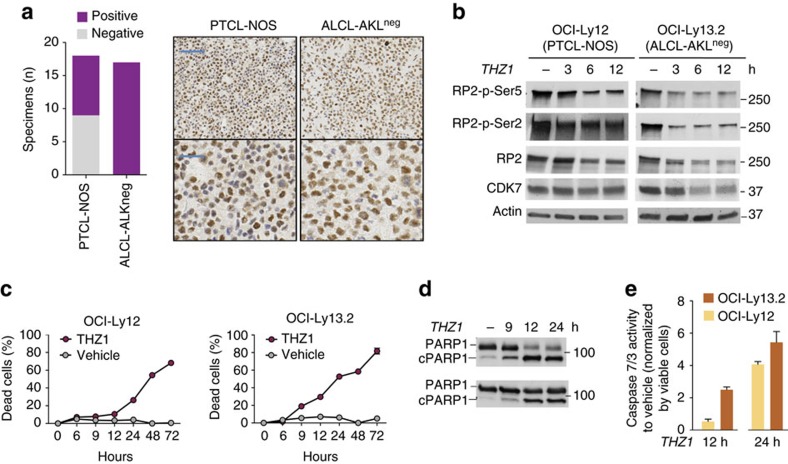
CDK7 is a survival factor in PTCL. (**a**) CDK7 protein expression by immunohistochemistry in PTCL-NOS (*n*=18) and ALCL-ALK^neg^ (*n*=17) cases. Representative microphotographs (upper pictures taken at 10 × , scale bar represents 100 μm and lower pictures at 40 × , scale bar represents 50 μm) are shown on the right. (**b**) Immunoblots of CTD phospho-Ser5 and phospho-Ser2 of RNA polymerase II (RP2) and total RNA polymerase II and CDK7 in PTCL cells treated with THZ1 for the indicated time points. (**c**) Proportion of dead cells in OCI-Ly12 and OCI-Ly13.2 cells treated with THZ1 500 nM for up to 72 h. (**d**) PARP1 and cleaved PARP1 in OCI-Ly12 and OCI-Ly13.2 cells treated with THZ1 500 nM for up to 24 h. (**e**) Caspases 7 and 3 activity in OCI-Ly12 and OCI-Ly13.2 cells treated with 500 nM of THZ1 for 12 and 24 h. Data is presented as fold-change in activity relative to vehicle as means with 95% CI for quintuplicates.

There are no targeted agents for the most common PTCL sub-types, PTCL-NOS and ALCL-ALK^neg^. To determine whether this subgroup of patients could benefit from a CDK7 targeting approach, we analysed the expression of CDK7 in 18 PTCL-NOS and 17 ALCL-ALK^neg^ cases. We found CDK7 nuclear expression in 50% and 100% of PTCL-NOS and ALCL-ALK^neg^ patients, respectively (Fig. 2a). In PTCL-NOS and ALCL-ALK^neg^ cell lines, CDK7 is also overexpressed compared with normal T cells from tonsils (Supplementary Fig. 2), suggesting a higher requirement for CDK7 in these PTCL subtypes. CDK12 that can be inhibited by THZ1 at higher concentrations is expressed at lower levels in PTCL cell lines (Supplementary Fig. 2). In addition to CDK7, the other two components of the CDK-activating kinase sub-unit of the TFIIH complex^18^,^19^, CCNH and MAT1A, are overexpressed in PTCL cells as well (Supplementary Fig. 2).

We therefore studied the mechanism of action of THZ1 in the PTCL-NOS cell line OCI-Ly12 and the ALCL-ALK^neg^ cell line OCI-Ly13.2. CDK7, associated to the TFIIH complex, phosphorylates RNA POL2 at the Ser5 during RNA transcription. Accordingly, THZ1 exposure induced a time-dependent decrease in the phosphorylation of RNA POL2 Ser5 (Fig. 2b) and RNA POL2 Ser2 (Fig. 2b). The mechanism leading to decrease in the phosphorylation of RNA POL2 Ser2, that unlikely involves a direct effect on CDK7, remains to be identified^17^. The CDK-activating kinase subunit has also been implicated in the activating phosphorylation of several cell-cycle kinases including CDK1, CDK2, CDK4 and CDK6 (ref. 20), we thus determined the effect of THZ1 on cell cycling. We found that THZ1 at 3, 6 and 9 h induces minimal perturbation of the cell cycle and, accordingly, almost no variations in the levels of cyclins A, B1, D1, E1 and E2 in OCI-Ly12 and OCI-Ly13.2 cells (Supplementary Fig. 3a,b). The main effect is a rapid induction of cell death in both cell lines (Fig. 2c) that is associated with PARP cleavage (Fig. 2d) and activation of caspases 7 and 3 (Fig. 2e); suggesting that CDK7 transcriptional activity could be required for survival of PTCL-NOS and ALCL-ALK^neg^ cells.

### THZ1 disassembles mutant STAT3 transcriptional complexes

**Figure 3:**
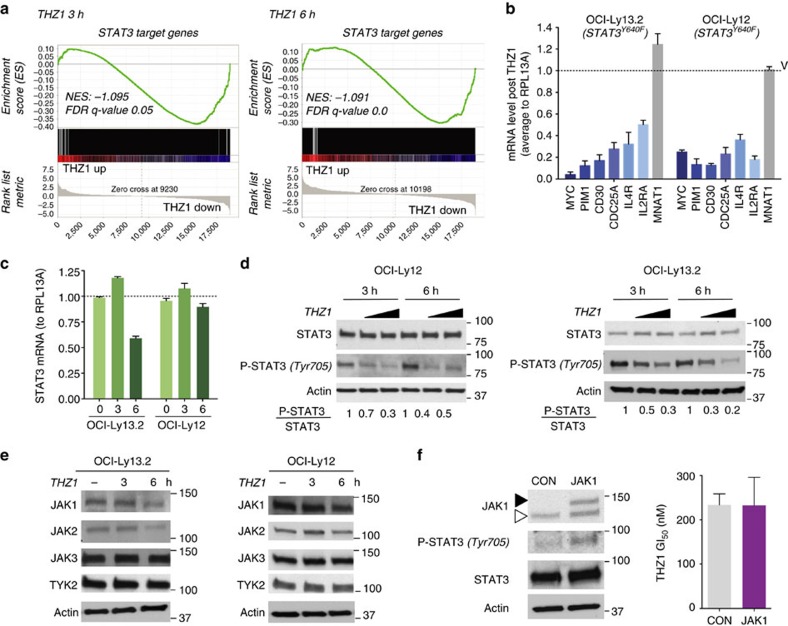
THZ1 inhibits STAT3^Y640F^ signalling. (**a**) Gene set enrichment analysis of STAT3 target genes^24^ using ranked gene expression changes after 500 nM THZ1 treatment for 3 h (left) and 6 h (right) in OCI-Ly13.2 cells. NES and FDR are indicated based on 5,000 permutations. (**b**) Transcript levels (to RPL13A) of the selected STAT3 target genes *MYC, PIM1, CD30, CDC25A, IL4R* and *IL2RA* and the negative control *MNAT1* after treatment of OCI-Ly13.2 and OCI-Ly12 cells with 500 nM THZ1 for 6 h. Data are presented as mean with 95% CI for triplicates. (**c**) Time-dependent effect of THZ1 500 nM in STAT3 transcript level in OCI-Ly12 and OCI-Ly13.2 cells. Data are presented as mean with 95% CI for triplicates. (**d**) Time and dose-dependent protein levels of STAT3 and phospho-STAT3 Tyr705 in OCI-Ly12 and OCI-Ly13.2 cells treated with THZ1 250 and 500 nM. Densitometry analysis is shown at the bottom. (**e**) Time-dependent effect of THZ1 500 nM in protein levels of the Tyr705 STAT3 kinases JAK1, JAK2, JAK3 and TYK2. (**f**) Effect on the GI_50_ of THZ1 in Jurkat cells transfected with an active JAK1 plasmid. Endogenous JAK1 is shown with white arrowhead and exogenous JAK1 is marked with black arrowhead. Data are presented as mean with 95% CI for triplicates.

CDK7 kinase inhibition reduces nascent transcription and increases the stability of existing mRNA^21^. We reasoned that THZ1 treatment would more greatly impact those transcripts that were actively transcribed in PTCL. Recent findings showed proliferation and survival programs in ALCL-ALK^neg^ and PTCL-NOS patients are driven by the presence of activating mutations on STAT3 or mutations involving other genes that secondarily activate STAT3 such as JAK1 or kinase fusions^22^. We therefore analysed our PTCL cell lines for the presence of known PTCL recurrent mutations in STAT3 (Y640F and D66I) and JAK1 (L910P). We found that OCI-Ly12 (PTCL-NOS) and OCI-Ly13.2 cells (ALCL-ALK^neg^) harbour an Y640F mutation in the SH2 domain of STAT3 that confers greater dimer stability and transcriptional activity^22^,^23^. To determine whether the STAT3 transcriptional program is susceptible to CDK7 inhibition, we first conducted RNA-sequencing in OCI-Ly13.2 cells treated with THZ1 500 nM (or vehicle) for 3 and 6 h in triplicates. We then obtained significantly changed transcripts (*q*-value <0.01; FDR, false discovery rate) and compared them with the target genes of STAT3 obtained from the STAT3 ChIP-seq of IL-6-stimulated T cells^24^. Gene set enrichment analysis showed a significant enrichment of STAT3 target genes among the genes downregulated after THZ1 treatment at 3 h (Normalized Enrichment Score, NES: −1.095, *q*-value 0.05, FDR) and 6 h (NES: −1.091, *q*-value 0.0, FDR) (Fig. 3a). We found several downregulated STAT3 targets relevant for PTCL survival such as *MYC, PIM1, MCL1, CD30, IL2RA, CDC25A, IL4R* and *MIR21* (Supplementary Data 1). We independently confirmed the downregulation of selected STAT3 target genes by qRT-PCR in OCI-Ly13.2 and OCI-Ly12 cells at 6 h (Fig. 3b). MNAT1 that is not a STAT3 target gene did not decrease with THZ1 (Fig. 3b). This suggests that CDK7 activity is necessary to sustain actively transcribed STAT3 target genes in PTCL.

To further elucidate the mechanism of CDK7 on maintaining the STAT3 transcriptional program, we analysed the effect of THZ1 on STAT3 expression. Upon THZ1 treatment, there was a decrease in STAT3 mRNA level only in OCI-Ly13.2 cells at 6 h (Fig. 3c) with no discernible impact in STAT3 protein levels (Fig. 3d). We therefore investigated the activation of STAT3. Mutant STAT3^Y640^ is constitutively phosphorylated at Tyr^705^ and is further activated by the IL-6/JAK pathway^25^. In contrast to total protein levels, we found that THZ1 induced a decrease in Tyr^705^ STAT3 phosphorylation between 30 to 70% as early as 3 h and between 50 to 80% at 6 h in OCI-Ly12 and OCI-Ly13.2 cells (Fig. 3d). There was no effect on STAT3 phosphorylation on Ser^727^ in any cell line (Supplementary Fig. 4). To determine whether CDK7 decreases STAT3 phosphorylation by downregulating the expression of kinases that transduce STAT3-activating signals, we analysed the expression of JAK1, JAK2, JAK3 and TYK2 after THZ1 treatment. CDK7 inhibition led to a modest downregulation of JAK1 expression at 6 h in both cell lines (Fig. 3e), likely insufficient to explain the decrease in STAT3 (Tyr^705^) phosphorylation. To further elucidate the role of JAK1, we analysed whether JAK1 over-activation could overcome the effect of THZ1. We engineered TCL/L Jurkat cells (non-mutated STAT3 and JAK1 genes) to express an active form of JAK1 and exposed them to IL-6 50 U ml^−1^ resulting in increased STAT3 phosphorylation (Fig. 3f). We determined the THZ1 GI_50_ in these cells (versus control plasmid transfected cells) and found no significant differences (Fig. 3f). Taken together, these results suggest that THZ1 decreases STAT3 phosphorylation through a JAK and TYK2 independent mechanism.

**Figure 4:**
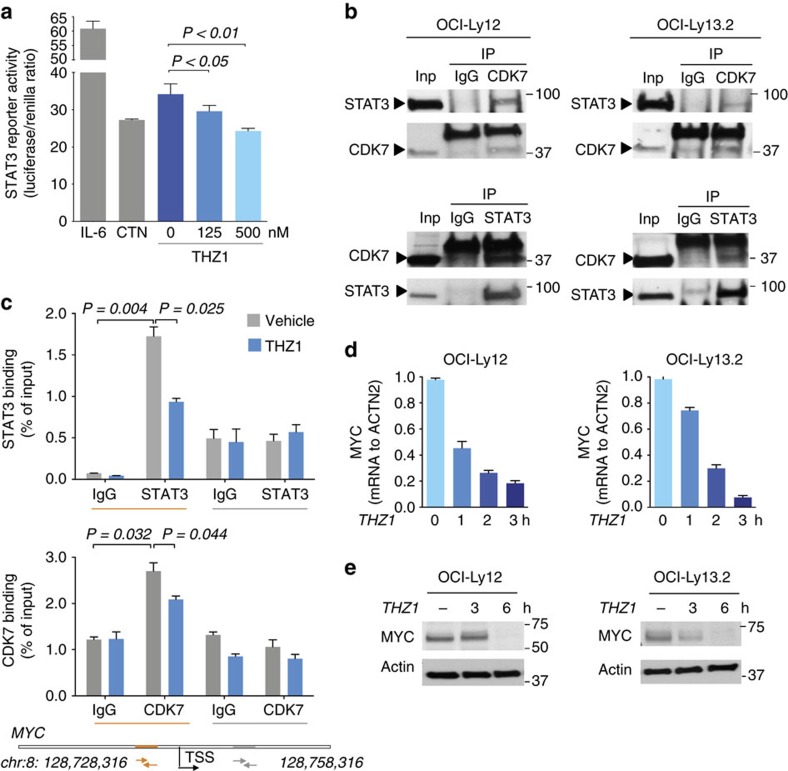
THZ1 unbinds mutant STAT3 from the *MYC* gene. (**a**) STAT3 reporter activity upon administration of IL-6 10 ng ml^−1^, the STAT3 inhibitor cryptotanshinone (CTN) 2.5 μM and THZ1 (vehicle, 125 and 500 nM). Data are presented as median with median absolute deviation for quintuplicates. *P*-values obtained from a Mann–Whitney test. (**b**) Reciprocal CDK7 and STAT3 co-immunoprecipitations in OCI-Ly12 and OCI-Ly13.2 cells. (**c**) CDK7 and STAT3 q-ChIP (IgG was used as control) on *MYC* gene in OCI-Ly13.2 cells baseline (vehicle, gray columns) and after 2 h of THZ1 500 nM (blue columns). Data presented as mean with standard error of the mean (SEM) for two independent replicates of triplicates. *P*-values obtained from a *T*-test. (**d**) MYC transcript levels in OCI-Ly12 and OCI-Ly13.2 cell lines upon THZ1 500 nM treatment for the indicated time points. Data are presented as mean with 95% CI for triplicates. *P*-values obtained from a *T*-test. (**e**) Myc protein levels in cells treated as in **d**.

Transcriptional complexes containing Tyr^705^ phosphorylated STAT3 recruit RNA POL2 to target genes^26^. These complexes remain assembled and associated to chromatin in continued presence of STAT3 inducers^26^. To directly assess the effect of THZ1 on STAT3 activation, we conducted a luciferase reporter assay in 293T cells transfected with *SOCS3* promoter containing STAT3 binding sites. After transfection, cells were treated with IL-6 10 ng ml^−1^ for 6 h (as positive control for STAT3 activity), cryptotanshinone 2.5 μM for 3 h (as positive control for inhibition of STAT3-dependent luciferase activity^27^) and THZ1 125 and 500 nM for 3 h. We found a significant inhibition of STAT3-dependent luciferase activity with both concentrations of THZ1 (*P*<0.05 and <0.01, *T*-test, respectively) to levels similar to cryptotanshinone 2.5 μM (Fig. 4a).

We then tested whether THZ1-induced RNA POL2 inhibition leads to mutant STAT3^Y640F^ chromatin dissociation and ultimately Tyr^705^ dephosphorylation. To determine the presence of CDK7 on STAT3^Y640F^ complexes, we conducted endogenous co-immunoprecipitations and found that STAT3^Y640F^ and CDK7 co-immunoprecipitated in reciprocal experiments in OCI-Ly12 and OCI-Ly13.2 cells (Fig. 4b). To ascertain their association with active chromatin, we conducted STAT3 Q-ChIP and CDK7 Q-ChIP on the STAT3 target gene *MYC* in OCI-Ly13.2 cells and found a significant enrichment of STAT3 and CDK7 at the promoter region of *MYC* that possesses a STAT consensus sequence (*P*=0.004 and *P*=0.032, respectively, *T*-test Fig. 4c). There was no significant enrichment at a downstream intronic region on *MYC* lacking a STAT consensus sequence (Fig. 4c). Treatment of OCI-Ly13.2 cells with THZ1 for 2 h significantly decreased STAT3 and to a lesser extent CDK7 peaks on the *MYC* gene (*P*=0.025 and *P*=0.044, respectively, *T*-test, Fig. 4c), suggesting that CDK7 activity is necessary for continued association of STAT3^Y640F^ at the chromatin of its target genes. In accordance, transcription of MYC decreased significantly after 3 h of THZ1 (Fig. 4d) followed by decreased levels of MYC protein (Fig. 4e) in OCI-Ly12 and OCI-Ly13.2 cells.

### THZ1 decreases the activity of STAT1 and STAT5

**Figure 5:**
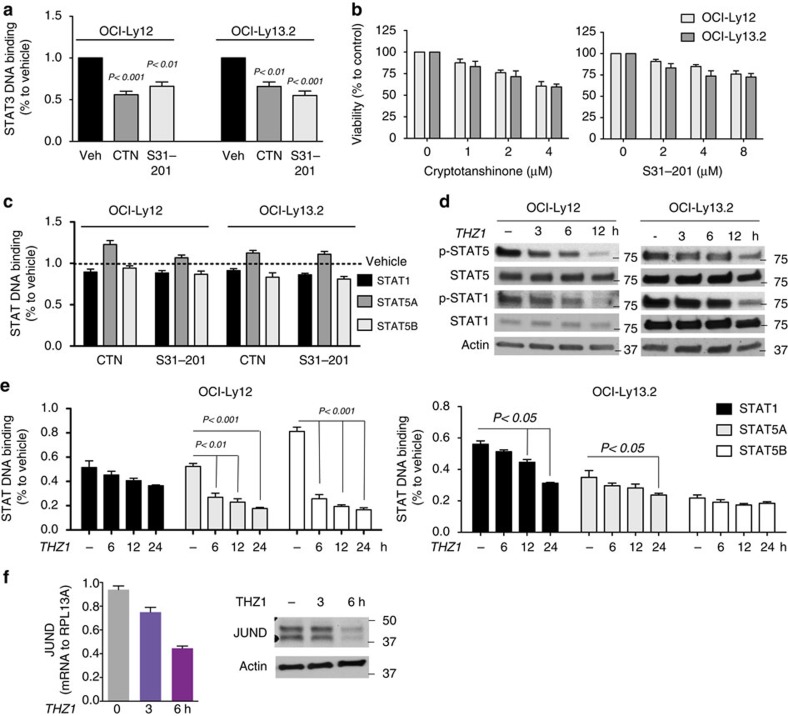
THZ1 inhibits STAT1 and STAT5 signalling. (**a**) Effect on STAT3 DNA binding activity of the STAT3 inhibitors cryptotanshinone (CTN) and S31-201 in STAT3 mutant cell lines OCI-Ly12 and OCI-Ly13.2. (**b**) Effect on cell viability at 48 h of the STAT3 inhibitors cryptotanshinone and S31-201 in OCI-Ly12 and OCI-Ly13.2 cells. (**c**) Activity of STAT1, STAT5a and STAT5b measured by DNA biding to a consensus sequence in nuclear extracts of OCI-Ly12 and OCI-Ly13.2 cells treated with cryptotanshinone (CTN) and S31-201. (**d**) Immunoblots of STAT5, STAT1, phospho-STAT5 and phospho-STAT1 (to actin) in PTCL cells treated with THZ1 500 nM for the indicated time points. (**e**) Activity of STAT1, STAT5a and STAT5b measured by DNA biding to a consensus sequence in nuclear extracts of OCI-Ly12 and OCI-Ly13.2 cells treated with 500 nM of THZ1 for the indicated time points. (**f**) JUND transcript (left) and protein (right) levels in OCI-Ly13.2 cells upon THZ1 500 nM treatment for the indicated time points. All data are presented as mean with 95% CI for triplicates. *P*-values obtained from *T*-test.

To determine what the extent of THZ1 anti-lymphoma activity is due to STAT3 inhibition, we treated OCI-Ly12 and OCI-Ly13.2 cells with selective STAT3 inhibitor compounds. Administration of S31-201, a STAT3 dimerization inhibitor, and cryptotanshinone, a STAT3 Tyr^705^ inhibitor, to OCI-Ly12 and OCI-Ly13.2 cells resulted in significant blunting of STAT3 DNA binding by 40–50% (Fig. 5a). However, both STAT3 selective inhibitors showed a modest decrease in cell proliferation and viability in OCI-Ly12 and OCI-Ly13.2 cells (Fig. 5b), contrasting with the effect of THZ1 in these cells. Because selective inhibition of STAT3 can induce compensatory on-target feedback mechanisms that may result in activation of other STATs and decreased anti-tumoural effect^4^, we investigated the activation of STAT1 and STAT5 upon selective STAT3 inhibition. Administration of S31-201 and cryptotanshinone resulted in maintained or increased levels of STAT1 and/or STAT5 binding activity in both cells lines (Fig. 5c). In contrast, THZ1 administration resulted in a simultaneous decrease of phospho-STAT1 Tyr^701^ and phospho-STAT5 Tyr^694^ in OCI-Ly12 and OCI-Ly13.2 cells at 12 h after compound administration (Fig. 5d). This was associated with significant decrease of STAT1, STAT5a and/or STAT5b DNA binding in OCI-Ly12 and OCI-Ly13.2 cells (Fig. 5e). Accordingly, transcript and protein levels of the STAT5 target oncogene *JUND* decreased upon THZ1 treatment in OCI-Ly13.2 cells (Fig. 5f). JUND transcript started to decrease as early as 3 h after THZ1, suggesting that decrease in gene transcription is the primary event preceding changes in STAT phosphorylation and DNA binding as previously demonstrated for STAT3. This is of therapeutic relevance since PTCL patients frequently exhibit transcriptional signatures associated to the activation of other STAT members like STAT1 and STAT5 (ref. 4), and STAT5B activating mutations have been described in PTCL subtypes^28^. In fact, despite the presence of the STAT3^Y640F^ mutation, OCI-Ly12 and OCI-Ly13.2 cells also showed baseline tyrosine phosphorylation of STAT1 and STAT5 (Fig. 5d), a setting that would likely induce resistance to specific JAK or STAT inhibitors^4^.

### THZ1 inhibition of STAT sensitizes PTCL cells to BH3 mimetics

Although BCL2 family proteins are frequently expressed in most PTCL subtypes^29^,^30^,^31^,^32^, the majority of the cell lines are relatively resistant to the BH3-mimetic drugs in clinical use obatoclax, ABT-737 and venetoclax (ABT-199) (Supplementary Fig. 5). The mechanism of action of BH3-mimetic agents depends, in part, on overcoming sequestration of pro-apoptotic BH3 proteins by pro-survival BH3 proteins (for example, BCL2, BCL-XL and MCL1) that will release the M.O.M.P. (mitochondrial outer membrane permeabilization) effectors BAX and BAK to induce apoptosis^33^. In this regard, obatoclax is a pan-BH3 mimetic, ABT-737 binds to high affinity to BCL2 and BCL-XL but not to MCL1 and venetoclax is BCL2 selective^34^.

**Figure 6:**
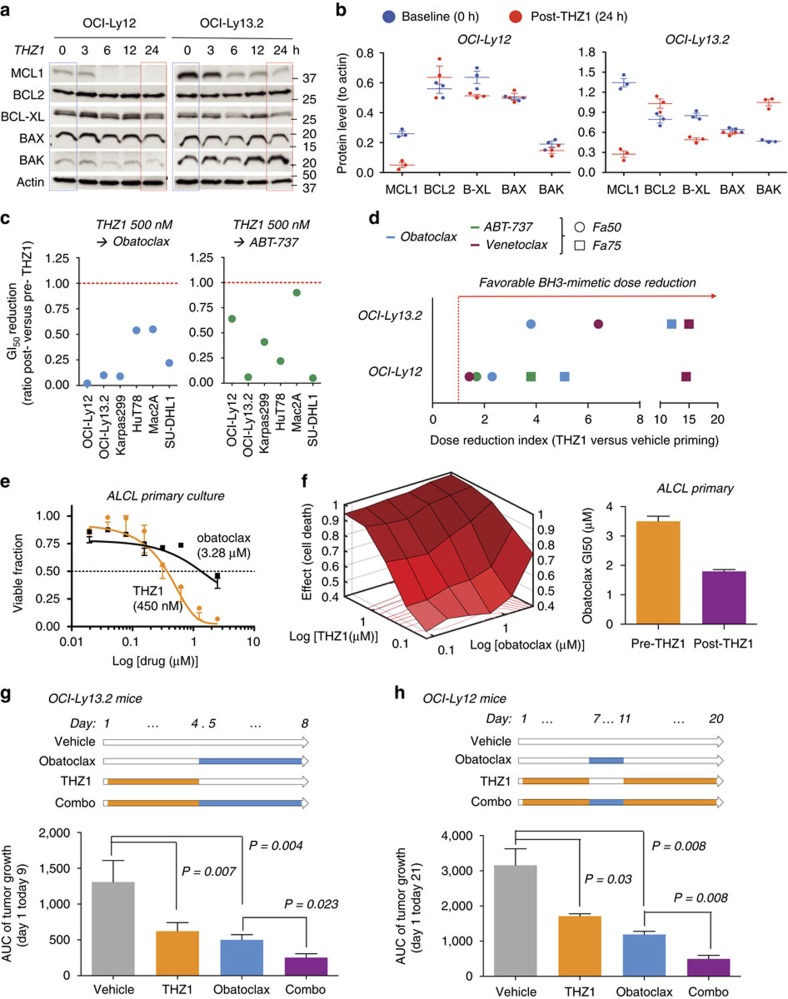
THZ1 sensitizes PTCL cells to BH3 mimetic drugs. (**a**) Time-dependent effect of THZ1 500 nM in protein levels of anti-apoptotic BH3 proteins MCL1, BCL2 and BCL-XL and BH3 effectors BAX and BAK. The blue square indicates baseline levels and red square indicate baseline levels in surviving cells at 24 h. (**b**) Protein levels as ratio of BH3 proteins to actin before (in blue) and after (in red) treatment with THZ1 for 24 h in triplicate experiments. Data are presented as mean with 95% CI. (**c**) Effect of THZ1 500 nM treatment for 24 h on the GI_50_ of obatoclax and ABT-737 expressed as ratio between post and pre-THZ1 in PTCL cell lines. Red dotted line at 1.00 indicates unchanged ratio and points below the line indicates a reduced GI_50_ after THZ1 at 48 h. (**d**) Dose-reduction index for BH3-mimetic drugs obatoclax, ABT-737 and venetoclax in the STAT3 mutant cell lines OCI-Ly12 and OCI-Ly13.2 after THZ1 priming for 24 h. The effects for fraction of cell killing 50% (Fa50) and 75% (Fa75%) are shown as circles or squares symbols, respectively. (**e**) Effect of THZ1 and obatoclax on the viability of an ALCL primary culture. The GI_50_ for each drug is shown between parentheses. (**f**) Response-surface analysis for the combination of eight concentrations of THZ1 and obatoclax on the viability of the primary ALCL culture. Darker shades of red indicate higher killing effect. Effect of THZ1 (at 1:5 ratio) on the GI_50_ of obatoclax. Data are presented as mean with 95% CI for triplicates. (**g**) Schedule of administration for *in vivo* treatment of OCI-Ly13.2 mice (top). Area under the curve (AUC) of tumour growth from day 1 to day 9 in OCI-Ly13.2 xenografted mice treated as shown in the schedule. (**h**) Schedule of administration for *in vivo* treatment of OCI-Ly12 mice (top). AUC of tumour growth from day 1 to day 21 in OCI-Ly12 xenografted mice treated as shown in the schedule. Data are presented as mean with 95% CI. *P-*values obtained from *T*-test.

Since pro-survival BCL2 family members are known STAT transcriptional targets, we hypothesized that THZ1 would alter this dynamic balance potentially sensitizing PTCLs to BH3 mimetics. We first investigated the expression of BCL2 family genes in PTCL cells that survived THZ1 administration. As expected from its transcriptional effect, exposing OCI-Ly12 and OCI-Ly13.2 cells to THZ1 500 nM induced a decrease in pro-survival BCL2, BCL-XL and MCL1 transcripts in both cell lines (Supplementary Fig. 6). This impacted the levels of short-lived proteins like MCL1 and BCL-XL, but not of BCL2 (Fig. 6a,b). There was also a simultaneous increase in amounts of the pro-apoptotic BAK in OCI-Ly13.2 (Fig. 6a,b). Overall, these THZ1-induced changes caused a shifted BH3 profiling (Fig. 6b) that could favour the activity of BH3-mimetic inhibitors in surviving cells. Accordingly, priming with THZ1 500 nM for 24 h decreased the GI_50_ of obatoclax and ABT-737 in PTCL cell lines with active STAT3^WT^ and STAT3^Y640F^ mutations (Fig. 6c). However THZ1 priming was insufficient to induce significant sensitization to the BCL2-specific drug venetoclax in all the PTCL cell lines but OCI-Ly12 (not shown).

Because BH3-mimetic drugs have different potency and *in vitro* antineoplastic effect (Supplementary Fig. 5) we determined the best THZ1 combinatorial ratio for the maximum killing effect conducting a response-surface analysis^35^ of the combination of 8 doses of THZ1 with 8 doses of obatoclax, ABT-737 and venetoclax in OCI-Ly12 and OCI-Ly13.2 cells. The ratios associated with higher anti-lymphoma effect were 1:5 and 1:7.5 for THZ1:obatoclax, 1:25 and 1:25 for THZ1:ABT-737, 1:1 and 1:25 for THZ1:venetoclax in OCI-Ly12 and OCI-Ly13.2 cells, respectively. Using these ratios, we pretreated OCI-Ly12 and OCI-Ly13.2 cells with six concentrations of THZ1 for 24 h (or vehicle) followed by six concentrations of obatoclax, ABT-737 and venetoclax (or vehicle) for additional 24 h and calculated the dose reduction index (DRI)^36^ for 50 and 75% killing effects (Fa50 and Fa75). We found that THZ1 priming increased the potency of obatoclax and venetoclax in OCI-Ly12 and OCI-Ly13.2 and ABT-737 in OCI-Ly13.2 for the Fa50 and Fa75 levels (Fig. 6d). For obatoclax, the increase in potency (at Fa50) by 2.3- and 3.8-fold in OCI-Ly12 and OCI-Ly13.2 cells (Fig. 6d) was sufficient to shift them into the *in vitro* sensitivity area for this drug (Supplementary Fig. 5). Taking together, the combination of THZ1 with the pan-BH3 inhibitor obatoclax resulted the most effective across PTCL cell lines.

We tested this combination in a primary ALCL culture system by exposing *ex vivo* isolated lymphoma cells to eight concentrations of THZ1 and obatoclax. The GI_50_s for obatoclax and THZ1 in the primary ALCL culture were 3.28 μM and 450 nM, respectively (Fig. 6e) which, comparing to PTCL cell lines, indicates sensitivity to THZ1 and resistance to obatoclax (Fig. 1c; Supplementary Fig. 5). To determine the optimal THZ1 combinatorial ratio for the maximum killing we conducted a response-surface analysis^35^ of the combination of eight doses of THZ1 with eight doses of obatoclax at several ratios (Fig. 6f). We found that THZ1 increased the potency of obatoclax (at 1:5 THZ1:obatoclax ratio) therefore reducing its GI_50_ (Fig. 6f).

We then tested the combination of THZ1 and obatoclax in mice xenograft models of OCI-Ly12 and OCI-Ly13.2 according to schedules shown in Fig. 6g,h. For both xenografts, we found a higher anti-lymphoma effect in the combination compared with each drug alone (Fig. 6g,h), without increased toxicity measured by body weight, necropsy (except for splenomegaly in THZ1 treated mice) and biochemical analysis (performed in OCI-Ly12 mice only) (Supplementary Table 2).

Overall, our data provides an approach for therapy of PTCLs with constitutive activation of STAT signalling pathways through targeting of CDK7 and, in combination, CDK7-dependent target genes such as BCL2-family proteins.

## Discussion

Here, we addressed an unmet therapeutic need by identifying CDK7 as a novel target for most common aggressive T-cell lymphomas, PTCL-NOS and ALCL-ALK^neg^. Using an unbiased FDA-approved drugs screen approach, we observed that PTCL cells were highly susceptible to inhibitors of RNA POL2-dependent transcription, proteasome and KDAC. Among the proteasome inhibitors, bortezomib has been tested in a phase II study in CTCL and PTCL patients with overall response rate of 67%, including one complete response^37^. Romidepsin, the most active KDAC in our screen across all PTCL subtypes, is FDA-approved for second line treatment of relapsed PTCL patients^13^; which exemplifies the validity of our screening approach. We thus focused on the transcription inhibitors, represented in the initial screening by actinomycin (and in 3 out of 4 cell lines also by plicamycin), known for blocking the progression of RNA POL2. Although actinomycin is a clinically active chemotherapy agent, its toxicity, especially myelosupression, limits its clinical use as single agent and in combination. Actinomycin causes significant downregulation of actively transcribed genes in malignant T cells^38^, an effect that can also be obtained by using the CDK7 inhibitors^39^. In fact, a recent gene expression profiling comparing actinomycin- and THZ1-treated neuroblastoma cells showed a significant correlation between the two drugs^15^. In our models, THZ1 downregulated STAT-signalling dependent actively transcribed genes required for the maintenance of the malignant phenotype such as *MYC, PIM1, MCL1, CD30, IL2RA, CDC25A, IL4R* and *MIR21*. We demonstrated that this effect was partially a consequence of abrogating STAT3 activity in cells with STAT3^Y640F^ activating mutations.

A higher selectivity of THZ1 in PTCL compared with actinomycin or others CDK7 inhibitors could be due to the high expression of CDK7 in PTCLs and by the specific and covalent binding of THZ1 to CDK7 (ref. 9). In contrast, SNS-032, a non-covalent pan-CDK inhibitor (with CDK9 and CDK7 IC_50_=4 and 62 nM, respectively)^8^ was less potent and active across our PTCL cell line panel; in fact OCI-Ly12, the prototypical PTCL-NOS cell line, was resistant to this compound. On this regard, the inhibition of CDK12 and CDK13 might be contributing to some of the pro-apoptotic effects of THZ1 as recently demonstrated for selective inhibitors of these kinases^40^.

The non-oncogenic addiction of PTCL to CDK7 can be explained, in part, by the need for continued expression of oncogenes required for the malignant phenotype. Collectively, our data are consistent with the notion that CDK7 activity is required for the proper assembly of activated transcriptional complexes. Therefore, in PTCL cells THZ1 administration blunted the activity of STAT3 and other transcription factors leading to cell death. In surviving cells, the attenuation of STAT pathways increases their susceptibility to a specific targeted approach represented by obatoclax and other similar agents. Consequently, we showed that once CDK7 activity is suppressed, additional targeting of potential resistance mechanisms provides superior anti-lymphoma effect without increased toxicity to normal organs. Naturally, THZ1 suppression of the activity of other transcription factors could contribute to these combinatorial effects, such as its effects on MYC activity^15^. If anything, this adds to the potential appeal of using THZ1 to anchor combinatorial therapy for PTCL. We thus speculate that CDK7 inhibition (and possible of CDK12 and CDK13) could represent a cornerstone concept to build additional combinatorial regimens with targeted agents.

In conclusion, we identified CDK7 as a critical transcriptional regulator required to maintain the malignant phenotype in PTCL. We demonstrated that CDK7 activity is necessary for STAT-dependent transcription, an approach that could benefit patients with tumours that present interleukin- or mutation-driven activation of STAT programs like in PTCL^22^,^23^,^28^,^41^ or other malignances^42^,^43^. Importantly, we have identified the combination of THZ1 with BCL2 inhibitors as potential novel targeted agent-based therapeutic option for PTCL patients.

## Methods

### Cell lines

OCI-Ly12 and OCI-Ly13.2 (obtained from the Ontario Cancer Institute) and Karpas299 and SU-DHL1 (obtained from the DMSZ) were cultured in RPMI-1640 medium, supplemented with 10% FCS and 2 mM glutamine. Mac2A and HuT78 (obtained from the ATCC) were cultured with 20% FCS in RPMI-1640 or Iscove's modified Dulbecco's media (IMDM), respectively. 293T cells (obtained from the ATCC) were cultured in DMEM medium supplemented with 10% FCS. We conducted monthly tests for mycoplasm sp. and other contaminants and semi-annual cell identification by single-nucleotide polymorphism analysis.

### Compounds

The approved oncology drugs set (*n*=101) was provided (to L.C.) at no cost by the Developmental Therapeutics Program, Division of Cancer Treatment and Diagnosis, National Cancer Institute, National Institutes of Health (NIH) (Supplementary Table 1). Other compounds were obtained from Sigma: actinomycin and valproic acid sodium salt, from Selleck: SNS-032, carfilzomib (PR-171), romidepsin (depsipeptide), cryptotanshinone, S31-201, ABT-737, venetoclax (ABT-199) and obatoclax mesylate (GX15070), and from LC laboratories: bortezomib, panobinostat and vorinostat. The N. Gray laboratory provided THZ1 and THZ1-R.

### Compound testing

Cells were plated at appropriated concentrations to keep untreated cells growing in exponential rate over the complete drug exposure time (48–72 h) in 96-well plates. Each treatment condition was performed in at least three replicates. We used a fluorometric resazurin reduction method (CellTiter Blue, Promega) to determine cell viability. Resulting fluorescence (Ex_560 nM_/Em_590 nM_) was measured with a Synergy4 microplate reader (Biotek). Viable cells in all controls and randomly selected treated wells were also determined by trypan blue dye exclusion. Ratio of viable cells from the treatment group versus their vehicle control was calculated and converted into ‘effect'. Dose–effect curves were plotted to calculate drug concentrations that inhibit a percentage of cells (such as GI_50_ for 50% growth inhibition) using CompuSyn software (Biosoft). In combinatorial experiments, drugs were administered sequentially (THZ1→BH3-mimetic) in constant ratios and variable ratios and the effect on cell viability was determined as before. In addition, viable cells in all controls and treated wells were also determined by trypan blue dye exclusion. The best THZ1 combinatorial ratio for the maximum killing effect was determining by a response-surface analysis matrix^35^ of the combination of eight doses of each drug in the combination. Dose reduction index 36 was determined using the CompuSyn software.

### Caspase activity

Caspase 7 and 3 activity was determined using caspase-Glo 3/7 Assay (Promega, USA) following manufacturer's instructions. PTCL cell lines were treated in triplicate with vehicle and THZ1 500 nM and evaluated at 12 and 24 h in viable cells determined by trypan blue dye exclusion and CellTiter Blue (Promega) in parallel experiments. Luminescence was measured using the Synergy4 microplate reader (BioTek).

### Immunoblotting

Cell lysates were prepared using buffer containing 50 mM Tris pH 7.4, 150 mM NaCl, 1% NP-40, 0.25% sodium deoxycholate and 0.1% SDS with of protease inhibitor cocktail (Roche) and phosphatase inhibitor (PhosStop, Roche). Primary antibodies were used at 1:1,000 dilutions unless otherwise specified. Primary antibodies used were: rabbit anti-CDK7 (Cell Signaling Technology: CST, 2090), rabbit anti-RNA Pol-2 (N-20, Santa Cruz Biotechnology: SC-899), rabbit anti-phospho Serine-2 RNA Pol-2 (Bethyl Lab; A300-654A), rabbit anti-phospho Serine-5 RNA Pol-2 and (Bethyl Lab; A300-655A), rabbit anti-PARP-1 (H-250, SC-7150), rabbit anti-STAT3 (CST 9132), rabbit anti-phospho-STAT3 (Tyr705, CST 9131), rabbit anti-phospho-STAT3 (Ser727, CST 9134), rabbit anti-STAT1 (CST 9175), rabbit anti-phospho-STAT1 (Tyr701, CST 9167), rabbit anti-STAT5 (CST 9363), rabbit anti-phospho-STAT5 (Tyr694, CST 9359), rabbit anti-JAK1 (CST 3344), rabbit anti-JAK2 (CST 3230), rabbit anti-JAK3 (D1H3) (CST 8827), mouse anti-MYC (9E10, SC-40), mouse anti-MCL1 (22, SC-12756), mouse anti-BCL2 (C-2, SC-7382), rabbit anti-BCL-XL (54H6, CST 2764), rabbit anti-BAX (CST 2772), rabbit anti-BAK (CST 3814), rabbit anti-JUND (CST 5000), rabbit anti-TYK2 (SC-169), rabbit anti-Cyclin D1 (CST 2978), rabbit anti-Cyclin E1 (CST 4129), rabbit anti-Cyclin E2 (CST 4132), rabbit anti-Cyclin B1 (CST 4138), mouse anti-Cyclin A2 (CST 4656), and mouse anti-β-actin (Sigma-Aldrich; AC-74). The secondary antibodies used were goat anti-mouse (SC-2005) and anti-rabbit IgG-HRP conjugated (SC-2004).

### PTCL tissues samples and immunohistochemistry

samples considered pathology discards from primary PTCLs were obtained at the time of diagnosis from the University of Turin (Azienda Ospedaliera Universitaria, Citta' della Salute e della Scienza of Torino, Italy), the Albert Einstein College of Medicine (Bronx, New York) and Weill Cornell Medicine. Diagnoses were assigned according to the WHO classification by at least two experienced pathologists. Informed consents were obtained following the recommendations of local ethical committees. Representative formalin-fixed tumour sections and/or tissue microarrays were processed for CDK7 immunohistochemistry on a semi-automated staining apparatus using a rabbit anti-CDK7 antibody (CST-2090).

### RT-PCR and STAT3 and JAK1 mutation analysis

RNA was isolated from cell lines by TRIzol reagent (Invitrogen) and amount measured using the Nanodrop 1,000 spectrophotometer. Equal amounts of RNA were converted to cDNA using Verso cDNA Synthesis Kit (Thermo scientific). qRT-PCR was performed in 384-well plates using a 7,900 HT fast real-time PCR system (Applied Biosystems). Fold change in gene expression was calculated using the ^ΔΔ^CT method. Presence of STAT3 (Y640F and D66I) and JAK1 (L910P) mutations in DNA from PTCL cell lines and normal T cells were determined as previously described^22^.

### Co-immunoprecipitation

PTCL cell lysates from 2 × 10^7^ cells were prepared in buffer containing 25 mM Tris pH 8.0, 150 mM NaCl, 1 mM EDTA, 0,5% Triton X-100, protease inhibitors and pre-cleared with protein-A agarose beads. Lysates were incubated with 2 μg of anti-STAT3 (SC-482) or anti-CDK7 (SC-7344) antibodies or IgG (Santa Cruz) control over night at 4°C. Protein-antibody complexes were precipitated Protein A agarose beads, extensively washed, loaded onto SDS-PAGE gel with SDS-PAGE loading buffer and immunoblotted as described above. Uncropped blots are shown as Supplementary Fig. 7.

### Q-ChIP

CDK7 and STAT3 Q-ChIPs were performed as previously described^44^. STAT3 and CDK7 pull-downs were done with the anti-STAT3 (SC-482) and anti-CDK7 (SC-7344) antibodies, respectively. IgG (Santa Cruz) was used a negative control. STAT3 binding site on MYC were analysed using the primers CTGTGTCTTCTGCACCAGGA and AGAAGGACACATGGCTGGAC. To amplify a negative control region on MYC we used: ACTATTCCTGCCTCCCTGCT and CCTGGGCAACAGAGCTAGA.

### STAT DNA-binding activity and STAT3 reporter

STAT activity was determined in nuclear pellets of PTCL cells using the STAT transcription factor assay kit (TransAM, Active Motif) following manufacturer's instructions. Protein concentration in nuclear extracts was measured using the BCA kit (Thermo scientific). Resultant absorbance at 450 nm that correlates with STAT binding to a consensus DNA sequence was read using the Synergy4 microplate reader (BioTek). STAT3-luciferase reporters based on SOCS3 promoter region and mutant controls were previously published^22^. Briefly, 293T cells were transfected in six-well plates using polyethylenimine (Polysiciences). The plasmids co-transfected for STAT3 reporter activity and for transfection normalization were pGL3-SOCS3-FireflyLuc and pGL3-RenLuc, respectively, at 5:1 ratio. After 24 h, cells were replated in 96-well plates for 24 h and then treated with compounds for 3 or 6 h including vehicle, THZ1 (125 and 500 nM), cryptotanshinone (2.5 μM) and IL-6 (10 ng ml^−1^). Firefly and renilla luciferase activities were measured following manufacturer's instructions (Dual-Luciferase Reporter Assay System, Promega) using the Synergy4 microplate reader (BioTek).

### RNA-sequencing

Ly13.2 cells were treated with 500 nM THZ1 or vehicle for 3 or 6 h in triplicate. Cells were pelleted and RNA was isolated with RNeasy Micro Kit (Qiagen). Genomic DNA was eliminated and RNA integrity was verified using the Agilent 2,100 Bioanalyzer (Agilent Technologies). RNA integrity number values were greater than 8 for all samples. Sequencing libraries were generated using the TruSeq RNA sample prep kit (Illumina). Libraries were cluster amplified and sequenced for 50 cycles using the Illumina HiSeq2500. Reads were aligned to human hg19 using STAR^45^, and gene expression values were calculated by counting the reads mapping uniquely to the union of all gene exons using HTseq-count^46^. Differentially expressed genes between paired samples were identified with the edgeR package^47^. Genes with a FDR *q*-value<0.05 were considered differentially expressed.

### Mice experiments

The Institutional Animal Care and Use Committee of Weill Cornell Medicine approved all the experiments involving mice. Six- to eight-weeks old male and female NOD-SCID (NOD.CB17-Prkdc^scid^/J) mice were obtained from Taconic farms. A cohort of mice was subcutaneously injected in the flank with OCI-Ly12 lymphoma tissues or OCI-Ly13.2 cells in Matrigel (BD biosciences). When xenografts reached a palpable size (approximately 50–75 mm^3^), mice were randomized into two treatment arms to receive THZ-1 (10 mg kg^−1^ day^−1^) or vehicle (10% DMSO and 90% dextrose 5% in water) by intraperitoneal injection, followed later by another randomization into two treatment arms to receive obatoclax (2 mg kg^−1^ day^−1^) or vehicle (30% PEG300, 5% Tween-80 and 65% dextrose 5% in water) by intraperitoneal injection. Tumour volume was measured every other day for the duration of the experiment and the area under the tumour growth curve (AUC) was calculated with the software GraphPad PRISM 4.0 (GraphPad Software). The mice were then weighed every other day. All mice were sacrificed by cervical dislocation under anesthesia. At the end of the experiment, tumour and other tissues (including blood) were harvested, weighed and macroscopically examined for signs of tissue damage.

### Statistical analysis

Means (± 95% confidence interval) or medians (with median absolute deviation) of the different experimental groups were analysed for statistical significance with the software GraphPad PRISM 4.0 (GraphPad Software), using two-tailed *T*-test or Mann–Whitney test for parametric and non-parametric datasets, respectively. Differences were considered significant if *P*<0.05.

### Data availability

all relevant data are available from the authors. RNA-sequencing of Ly13.2 cells are available in Gene Expression Omnibus with the accession number GSE79955.

## Additional information

**How to cite this article:** Cayrol, F. *et al*. THZ1 targeting CDK7 suppresses STAT transcriptional activity and sensitizes T-cell lymphomas to BCL2 inhibitors. *Nat. Commun.* **8,** 14290 doi: 10.1038/ncomms14290 (2017).

**Publisher's note:** Springer Nature remains neutral with regard to jurisdictional claims in published maps and institutional affiliations.

## Supplementary Material

Supplementary Information Supplementary Figures and Supplementary Tables

Supplementary Data 1 RNA-sequencing of Ly13.2 cells treated with THZ1 for 3 h.
