# Corrigendum: THZ1 targeting CDK7 suppresses STAT transcriptional activity and sensitizes T-cell lymphomas to BCL2 inhibitors

**DOI:** 10.1038/ncomms14747

**Published:** 2017-02-20

**Authors:** Florencia Cayrol, Pannee Praditsuktavorn, Tharu M. Fernando, Nicholas Kwiatkowski, Rossella Marullo, M. Nieves Calvo-Vidal, Jude Phillip, Benet Pera, Shao Ning Yang, Kaipol Takpradit, Lidia Roman, Marcello Gaudiano, Ramona Crescenzo, Jia Ruan, Giorgio Inghirami, Tinghu Zhang, Graciela Cremaschi, Nathanael S Gray, Leandro Cerchietti

Nature Communications 8: Article number: 14290; DOI: 10.1038/ncomms14290 (2017); Published: 01 30 2017; Updated: 02 20 2017

The original version of this Article contained an error in the spelling of the author Rossella Marullo, which was incorrectly given as Rosella Marullo. This has now been corrected in both the PDF and HTML versions of the Article.

