# Supplementary material for: THZ1 targeting CDK7 suppresses STAT transcriptional activity and sensitizes T-cell lymphomas to BCL2 inhibitors
Source: Nat Commun. 2017 Jan 30;8:14290. doi: 10.1038/ncomms14290 (PMC5290269; doi:10.1038/ncomms14290)
Supplement: Supplementary Information — Supplementary Figures and Supplementary Tables [file ncomms14290-s1.pdf]

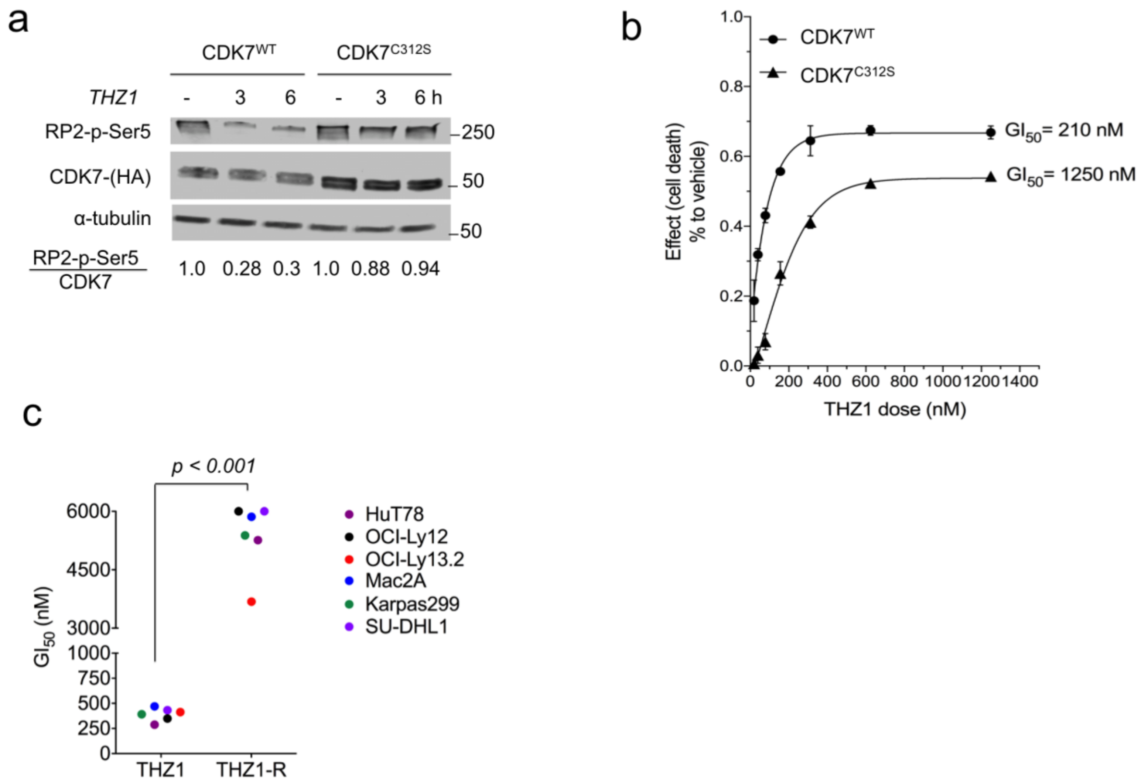

Supplementary Figure 1. (a) Effect of THZ1 500 nM at the indicated time-points on the phosphorylation of RNA-polymerase II at Ser5 in 293T cells transfected with $CDK7^{WT}$ and the $CDK7^{C312S}$ mutant. The densitometry (to CDK7) is shown at the bottom. **(b)** Dose-effect curve of THZ1 in 293T cells transfected with $CDK7^{WT}$ and the $CDK7^{C312S}$ mutant. The GI_{50} value is shown next to each curve. **(c)** GI_{50} for THZ1 and THZ1-R (a THZ1 analog with no significant inhibitory activity of CDK7) in our panel of PTCL cell lines. $P < 0.001$ (T-test).

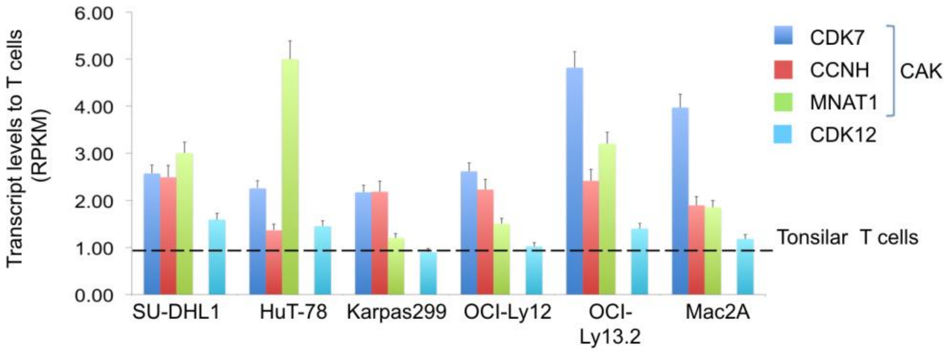

Supplementary Figure 2. Expression of the CAK components CDK7, CCNH and MNAT1, and CDK12 in PTCL cell lines compared tonsillar T-cells (dashed line). Data is presented as mean with 95% CI for triplicates.

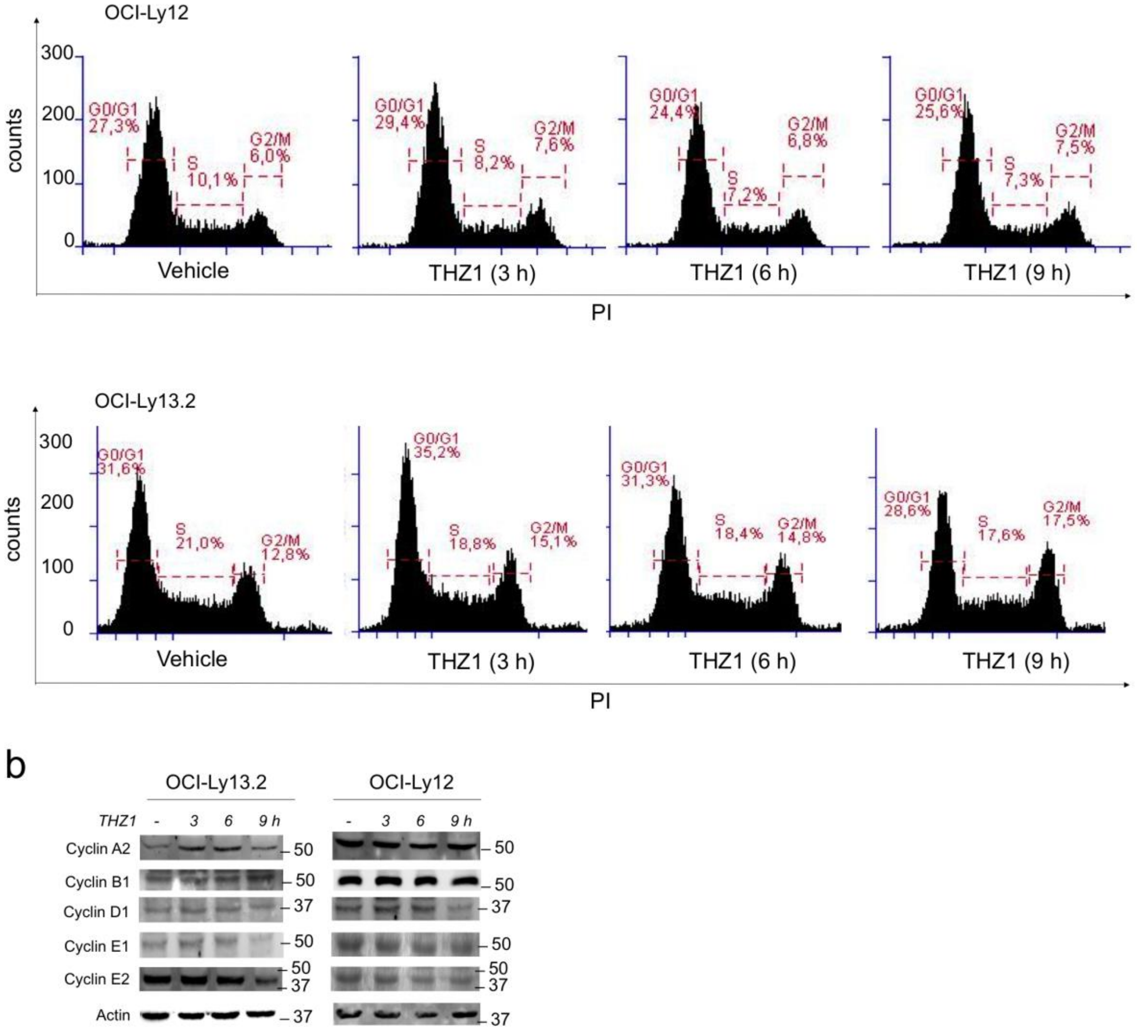

Supplementary Figure 3. (a) Cell cycle distribution of OCI-Ly12 (top) and OCI-Ly13.2 (bottom) cells treated with THZ1 500 nM for the indicated time points. **(b)** Protein levels of cyclin A, B1, D1, E1 and E2 in the cells treated as in (a).

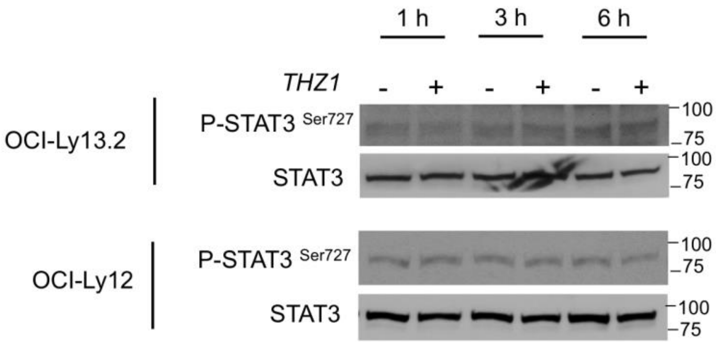

Supplementary Figure 4. Expression of STAT3 and Ser727 phosphorylated STAT3 in OCI-Ly12 and OCI-Ly13.2 cells treated with THZ1 for the indicated time points.

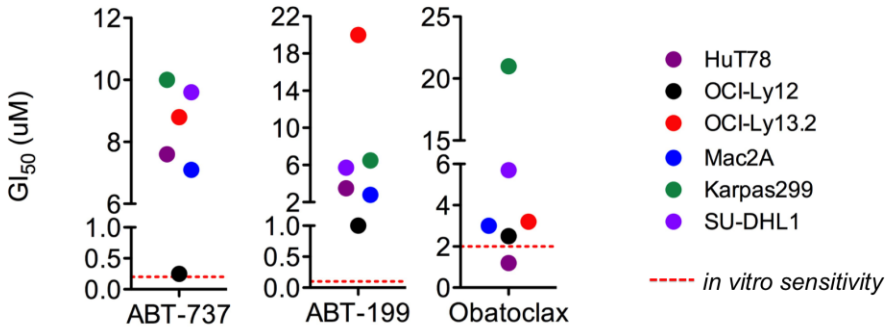

Supplementary Figure 5. GI_{50} of the BH3-mimetics ABT-737, ABT-199 (venetoclax) and obatoclax in the panel of 6 PTCL cell lines. The dashed line indicates the conservative value of *in vitro* sensitivity for “sensitive” cell lines according to published data in hematological and solid malignancies.

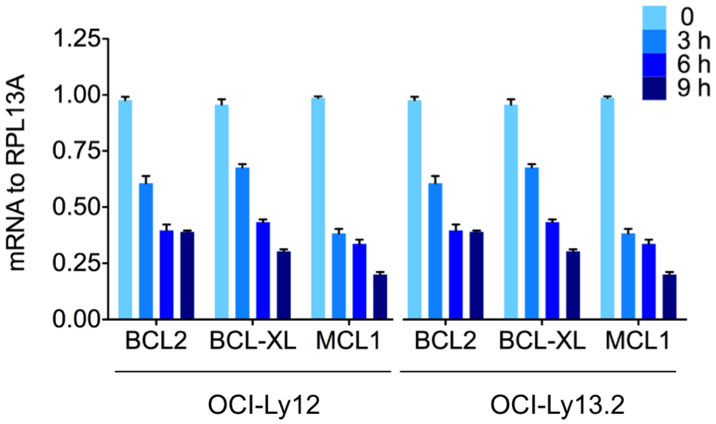

Supplementary Figure 6. Transcript levels of BCL2, BCL-XL and MCL1 in OCI-Ly12 and OCI-Ly13.2 cells treated with THZ1 500 nM for the indicated time points. Data presented as mean with 95% CI for triplicates.

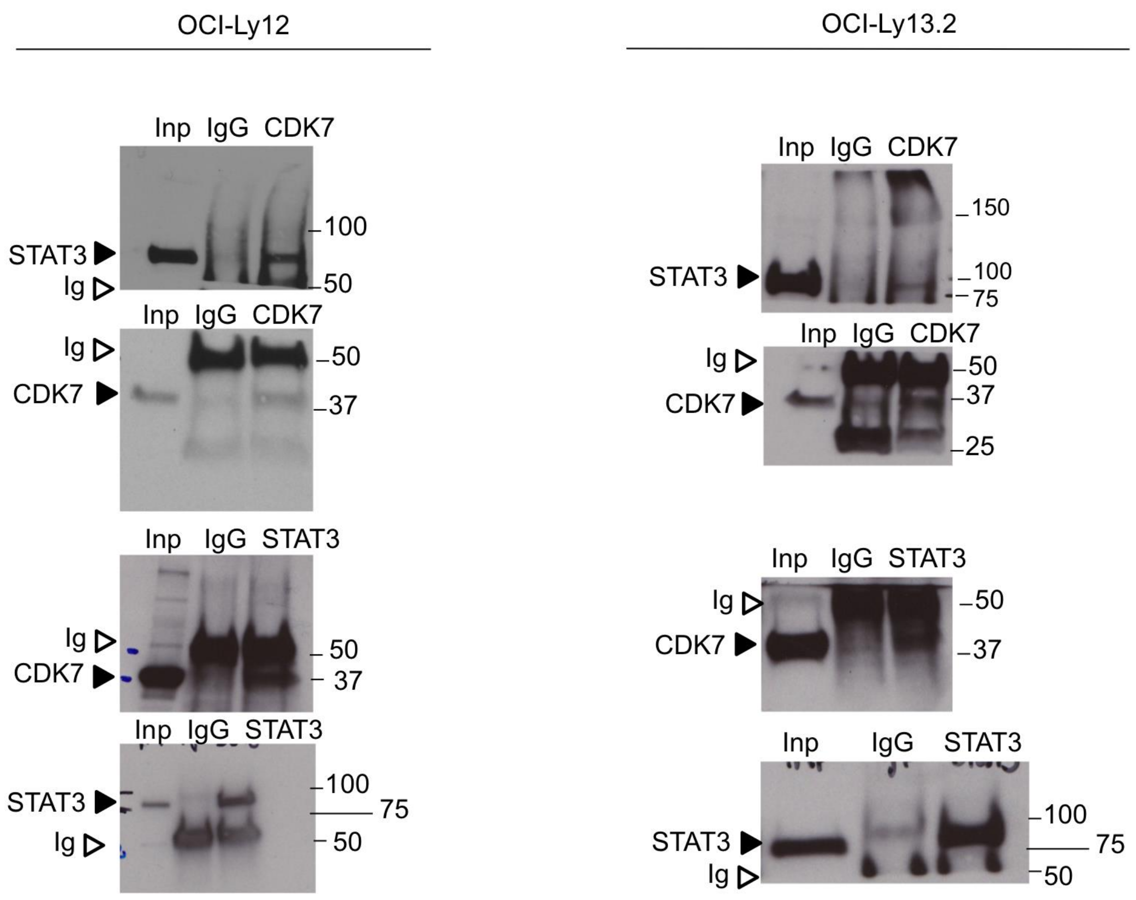

Supplementary Figure 7. Uncropped scans of the CDK7 and STAT3 co-immunoprecipitation experiments in OCI-Ly12 and OCI-Ly13.2 cells.

Supplementary Table 1. Compounds used in the screening and their activity showed according to the selected cut-offs.

Estimated GI_{50} in OCI-Ly12 primary screening				
**< 100 nM**	**< 1µM**	**< 10 µM**	**> 10 µM**	
Romidepsin	Teniposide	Paclitaxel	Lomustine: CCNU	Tretinoin
Bortezomib	Topotecan	Valrubicin	Floxuridine	Imiquimod
Carfilzomib	Vincristine Sulfate	Uracil mustard	Methotrexate	Everolimus
Actinomycin	Vinblastine Sulfate	Daunorubicin HCL	Mitotane	Zoledronic Acid
	Cabazitaxel	Doxorubicin HCL	Chlorambucin	Sirolimus
	Plicamycin	Mitomycin C	Vandetanib	Pralatrexate
	Gemcitabine	Mechlorethamine	Thiotepa	Abiraterone
	Clofarabine	Vorinostat	Letrozole	Allopurinol
	Mitoxantrone	Nilotinib	Mercaptopurine	Altretamine
	Docetaxel	Vinorelbin Tartrate	Axitinib	Amifostine
	Ixabepilone	Ara-C	Decitabine	Arsenic Trioxide
		Cladribine	Oxaliplatin	Bendamustine HCL
		Triethylen melamine	Vismodegib	Busulfan
		Etoposide	Anastrozole	Carmustine
		Azacitidine	Lenalidomide	Celecoxib
		Irinotecan HCL	Dexrazoxane HCL	Cisplatin
		Sorafenib	Dacarbazine	Cyclophosphamide
		Quinacrine	Procarbazine HCL	Erlotinib HCL
		Crizotinib	Nelarabine	Estramustine
		Thioguanine	Exemestane	Gefitinib
		Sunitinib Malate	Carboplatin	Ifosfamide
		Bleomycin	Hydroxyurea	Imatinib
			Aminolevulin Acid	Melphalan
			Streptozocin	Methoxsalen
			5-FU	Mitotane o,p'-DDD
			Megestrol acetate	Pipobroman
			Premetrexed Disodium	Pozapanib HCL
			Dasatinib	Raloxifene
			Thalidomide	Temozolomide
			Pentostatin	Vemurafenib
			Capecitabine	Bulsulfan
			Lapatinib Ditosylate	Fludarabine Phosphate

Supplementary Table 2. Serum biochemistry values from OCI-Ly12 mice treated with vehicle, obatoclax, THZ1 or their combination

	Vehicle	Obatoclax	THZ-1	Combination
RBC (M/uL)	11.0	12.5	12.1	11.6
HGB (g/dL)	15.9	17.2	16.6	15.8
HCT (%)	51.7	57.5	55.6	52.5
MCV (fL)	46.9	46.2	45.9	45.3
MCH (pg)	14.4	13.8	13.7	13.7
MCHC (g/dL)	30.7	29.9	29.9	30.2
RDW-SD (fL)	32.1	32.0	31.5	32.0
RDW-CV (%)	27.6	29.8	29.1	29.2
RET# (K/uL)	402.5	331.4	534.3	498.1
RET (%)	3.7	2.7	4.4	4.3
PLT (K/uL)	1417.5	936.7	1176.6	1285.0
PDW (fL)	10.8	10.0	9.9	9.3
WBC# (K/uL)	4.3	8.7	7.0	8.2
NEUT# (K/uL)	2.6	6.0	4.1	6.0
LYMPH# (K/uL)	0.8	1.5	1.4	1.0
MONO# (K/uL)	0.4	1.0	1.3	0.9
EO# (K/uL)	0.3	0.2	0.3	0.3
BASO# (K/uL)	0.1	0.0	0.0	0.0
NEUT (%)	62.3	56.8	58.2	66.8
LYMPH (%)	19.7	20.9	19.0	15.2
MONO (%)	10.3	15.4	18.3	14.0
EO (%)	6.7	6.8	4.5	3.8
BASO (%)	1.1	0.1	0.0	0.2
NEUT# (K/uL)	3.0	6.9	4.7	6.2
BANDS (%)	0.0	0.2	0.2	0.2
LYMPH# (K/uL)	1.0	1.1	1.0	1.1
MONO# (K/uL)	0.2	0.3	0.4	0.2
EO# (K/uL)	0.0	0.0	0.1	0.3
BASO# (K/uL)	0.0	0.0	0.0	0.0
BUN (mg/dL)	24.7	30.7	25.2	22.3
CREA (mg/dL)	0.2	0.3	0.2	0.2
ALP (U/L)	23.3	23.3	14.8	22.7
ALT (U/L)	450.3	108.7	222.2	289.0
AST (U/L)	492.0	402.0	459.6	539.0
GGT (U/L)	0.0	0.0	0.0	0.0
TBIL (mg/dL)	0.9	0.6	0.5	0.6
DBIL (mg/dL)	0.0	0.0	0.0	0.0
IBIL (mg/dL)	0.9	0.6	0.5	0.6
TP (g/dL)	6.6	5.9	6.1	6.1
ALB (g/dL)	3.4	3.0	3.0	3.0
GLOB (g/dL)	3.2	2.9	3.1	3.1
P (mg/dL)	11.6	13.8	9.3	9.3
Ca (mg/dL)	9.8	9.9	8.5	9.0
GLU (mg/dL)	185.3	147.0	169.8	188.0
CHOL (mg/dL)	186.8	172.8	145.2	138.8
TRIG (mg/dL)	319.0	179.0	234.6	161.8
CK (U/L)	464.3	1158.0	565.8	1021.3
